# Similarity Analysis of *Klebsiella pneumoniae* Producing Carbapenemases Isolated from UTI and Other Infections

**DOI:** 10.3390/antibiotics12071224

**Published:** 2023-07-24

**Authors:** Agata Pruss, Paweł Kwiatkowski, Monika Sienkiewicz, Helena Masiuk, Agnieszka Łapińska, Barbara Kot, Zuzanna Kilczewska, Stefania Giedrys-Kalemba, Barbara Dołęgowska

**Affiliations:** 1 Department of Laboratory Medicine, Pomeranian Medical University in Szczecin, Powstańców Wielkopolskich 72, 70-111 Szczecin, Poland; aga.lapinska@gmail.com (A.Ł.); kilczewskaz@gmail.com (Z.K.); barbara.dolegowska@pum.edu.pl (B.D.); 2 Department of Diagnostic Immunology, Pomeranian Medical University in Szczecin, Powstańców Wielkopolskich 72, 70-111 Szczecin, Poland; pawel.kwiatkowski@pum.edu.pl; 3 Department of Pharmaceutical Microbiology and Microbiological Diagnostic, Medical University of Lodz, Muszynskiego St. 1, 90-151 Lodz, Poland; monika.sienkiewicz@umed.lodz.pl; 4 Department of Medical Microbiology, Pomeranian Medical University in Szczecin, Powstańców Wielkopolskich 72, 70-111 Szczecin, Poland; helena.masiuk@pum.edu.pl (H.M.);; 5 Institute of Biological Sciences, Faculty of Exact and Natural Sciences, Siedlce University of Natural Sciences and Humanities, 14 Bolesława Prusa Str., 08-110 Siedlce, Poland; barbara.kot@uph.edu.pl

**Keywords:** carbapenemases, infections, PFGE, epidemiological investigation, *Klebsiella pneumoniae*

## Abstract

*Klebsiella pneumoniae* is an important opportunistic pathogen responsible for severe infections, mainly urinary tract infections (UTIs) and pneumonia. Hospital epidemic infections caused by multiresistant strains of carbapenemase-producing *K. pneumoniae* are the most concerning. NDM-producing strains are resistant to a wide range of antibiotics and have become the most significant threat. Determining the natural reservoirs and routes of infections is essential to end hospital outbreaks. Understanding the relatedness of *K. pneumoniae* strains is essential to determine the range and nature of the infection. The study compared phylogenetic relatedness between multiresistant *K. pneumoniae* strains isolated from hospitalized patients. Susceptibility to drugs and mechanisms of resistance were confirmed using phenotypic methods. PFGE was used to analyze the relatedness between strains. We analyzed 69 *K. pneumoniae* strains from various healthcare units. The isolates were mainly identified from urine. Strains were resistant to β-lactam antibiotics with β-lactamase inhibitors, cephalosporins, and quinolones. Their susceptibility to aminoglycosides and carbapenem antibiotics was diverse. Most of the isolated strains produced New Delhi metallo-ß-lactamase (NDM). Although *K. pneumoniae* strains were classified into several genotype clusters, closely related isolates were confirmed in the same hospital’s wards, and in two hospitals in the same province.

## 1. Introduction

*Klebsiella pneumoniae* was first identified by German bacteriologist Carl Friedländer in 1882 in a patient who died of pneumonia [1]. Today, it is one of the most commonly isolated microorganisms, causing hospital-acquired infections worldwide. Resistant to most commonly available antibiotics, it has become a “superbug” and thus a serious threat to public health [2]. The proliferation of plasmids encoding resistance mechanisms, especially carbapenemases, and the resulting antibiotic resistance have made *K. pneumoniae* a global healthcare challenge. The genetic diversity it exhibits tremendously impacts its pathogenic potential [3]. The bacterium settles in humans’ gastrointestinal tract and upper respiratory tract without showing any signs of infection [4]. From there, it can enter various tissues and cause dangerous infections. It leads to pneumonia, urinary tract infections, bacteremia leading to sepsis, soft tissue infections, liver abscesses, and related complications [5]. Initially, *K. pneumoniae* is the cause of severe infections in immunocompromised individuals, such as newborns, elderly individuals, and hospitalized individuals. Currently, hypervirulent strains of the bacteria have spread to the population of young, healthy people without immune deficiencies [6].

*K. pneumoniae* is a microorganism commonly found in the environment [7]. However, the most significant risk is its presence in hospitals, where the microorganism spreads and acquires new genes encoding pathogens or is responsible for drug resistance [3]. One of the reasons for the persistence of bacteria in healthcare facilities is the asymptomatic colonization of staff and patients, especially those hospitalized for long periods. Asymptomatic carriers cause continuous transmission of the pathogen, and the lack of control over its spread leads to numerous hospital outbreaks [4]. In addition, the lack of attention to the observance of industrial hygiene in hospitals and the fact that *K. pneumoniae* can persist in the hands of healthcare workers for up to several hours facilitating colonization. Another problem is medical instruments and devices, such as tracheostomy tubes, catheters, or drains [8]. Its persistence on these surfaces allows it to produce a biofilm, further protecting it from the effects of antibiotics and disinfectants. Drug-resistant *K. pneumoniae* causes the most worrisome hospital outbreaks. Isolates that produce multiple antibiotic resistance mechanisms have the most remarkable ability to spread. One of these mechanisms is the production of effective carbapenemases, which determine resistance to the entire group of β-lactams. Strains producing these enzymes are often also resistant to other groups of drugs (aminoglycosides, quinolones, trimethoprim-sulfamethoxazole). Most often, intrapatient transmission occurs within a region [9]. Additionally, the spread of genetically identical strains between countries or continents is possible only because of the constant movement of people and incidents of hospitalization outside the home country. Nevertheless, the epidemiology of nosocomial infections is specific to particular regions and countries [10].

The study aimed to assess the phylogenetic similarity of multidrug-resistant *K. pneumoniae* strains isolated from hospital patients.

## 2. Results

### 2.1. K. pneumoniae Isolation Sites

The most significant number of *K. pneumoniae* strains was isolated from a urine swab (*n* = 32, 46.4%). There were 18.85% (*n* = 13) isolates from BAL, 14.5% (*n* = 10) from the rectum, and 8.7% (*n* = 6) from blood. The fewest strains came from wound (*n* = 5; 7.25%) and pus (*n* = 3; 4.3%) swabs.

### 2.2. Drug Susceptibility

All *K. pneumoniae* strains tested were resistant to amoxicillin with clavulanic acid, piperacillin with tazobactam, cefotaxime, cefepime, and ciprofloxacin. Isolates showed differential resistance to aminoglycosides; 53.6% were resistant to amikacin, while 82.6% were resistant to gentamicin. Although the production of carbapenemase enzymes was confirmed by the phenotypic method, the strains were not utterly resistant to carbapenems. A higher percentages were strains resistant to meropenem (98.5%) andimipenem (97.1%) (Table 1).

### 2.3. Production of Carbapenemases

**Figure 1:**
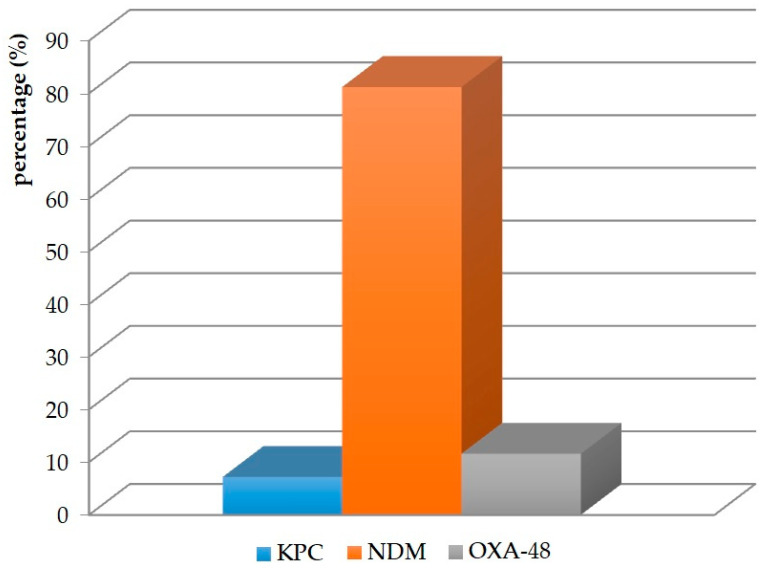
Carbapenemases produced by *K. pneumoniae* strains.

Of the 69 *K. pneumoniae* strains, the production of NDM-type metallo-β-lactamase was confirmed by phenotypic methods in 56 (81.2%). OXA-48 enzymes were present in eight(11.6%) strains, while KPC was present in five isolates (7.2%) (Figure 1).

### 2.4. Phylogenetic Similarity of K. pneumoniae Strains

**Figure 2:**
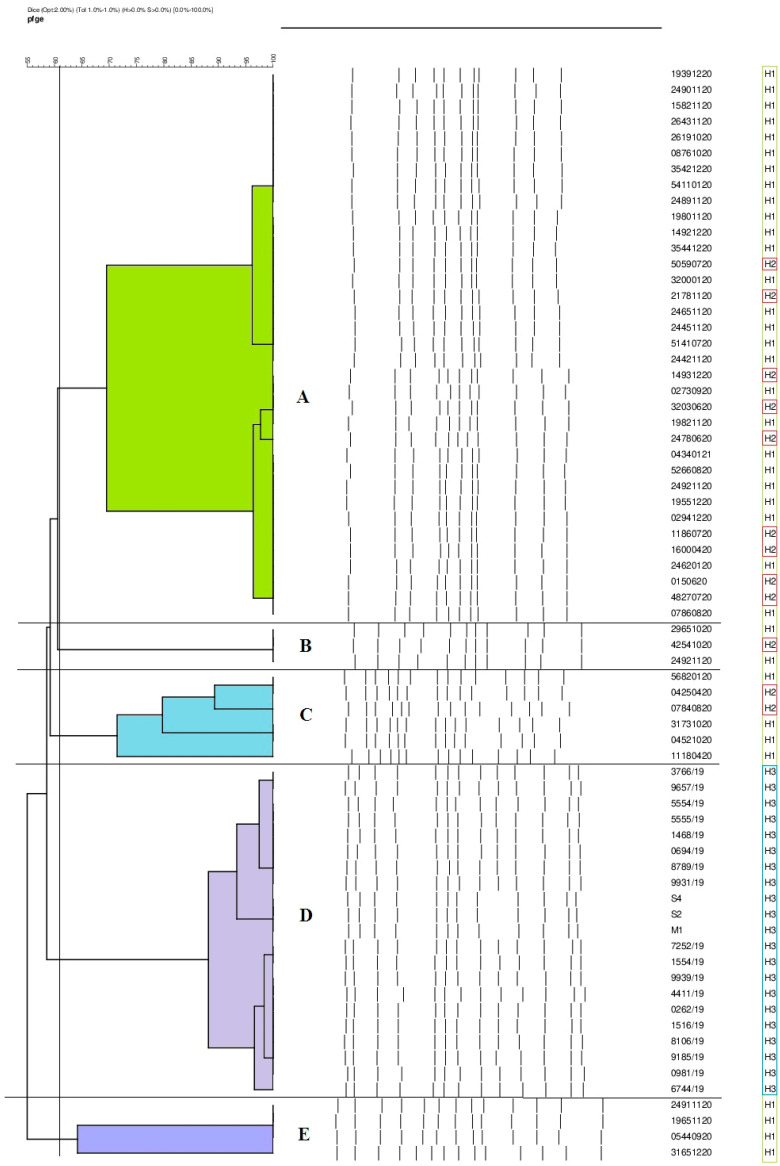
Dendrogram illustrating the genetic similarity of *K. pneumoniae* strains.

The phylogenetic similarity of the strains was analyzed by alternating field electrophoresis using a restriction enzyme. A similarity coefficient (SAB) value of 90% was used as the cutoff point. The strains were classified into five genotypes: A, B, C, D, and E. The most abundant genotype, designated profile A, contained 35 isolates. Some 21 strains were assigned to type D. The fewest isolates belonged to profiles B (3), C (6), and E (4). Strains isolated from patients hospitalized in Hospital No. 1 (H1) wards were classified into four genotypes. Of the 35 isolates from this hospital, 25 belonged to genotype A. Four strains each were assigned to profiles C and E. The fewest isolates (2) were classified as genotype B. Of the 13 strains from Hospital No. 2 (H2), 10 were assigned to genotype A. This facility also detected one isolate belonging to profile B and two isolates belonging to profile C. All the strains (21) from patients of Hospital No. 3 (H3) were classified only to genotype D. Among the strains analyzed, not a single strain was identified as unique. The results are shown in Table 2 and Figure 2.

## 3. Discussion

*K. pneumoniae* is an extremely dangerous commensal and opportunistic pathogen capable of causing severe nosocomial and nonhospital infections. The most significant problem for modern medicine is *K. pneumoniae* strains that produce carbapenemases capable of hydrolyzing β-lactam antibiotics, including carbapenems. The emergence of resistance to these antimicrobials contributes to high mortality rates among patients, as carbapenems are used as antibiotics of last resort to treat severe Gram-negative bacterial infections [11]. For this reason, *K. pneumoniae* was included in the list of “critical” pathogens published by the World Health Organization [12]. *K. pneumoniae* often causes epidemic outbreaks in healthcare facilities. The easy spread of microorganisms between wards is facilitated by the asymptomatic colonization of patients and hospital staff, and the microorganisms’ ability to persist on medical equipment [7]. This allows for the free transfer between strains of plasmids carrying genes encoding carbapenem-hydrolyzing enzymes. In addition, several resistance determinants can be present on a single plasmid, which contributes to the formation of multidrug-resistant *K. pneumoniae* strains that cause intrahospital epidemic outbreaks that are very difficult to contain [13]. A thorough analysis of how pathogen transmission occurs enables effective control of outbreaks. Molecular methods can determine the reservoirs of bacteria, their pathways of spread, and the affinity between strains. Continuous monitoring of “alert” pathogens, such as *K. pneumoniae*, is essential to avoid local and worldwide dangerous epidemics [14].

*K. pneumoniae* is the cause of UTIs, pneumonia, wound infections, and even blood infections. Infections occur primarily in hospitalized, severely ill, or immunocompromised patients. These microorganisms are also responsible for colonizing the gastrointestinal tract; the site from which they are most often isolated is the anus. The study by Aires-de-Sousa et al. showed that most isolates were obtained from rectal swabs (43.5%) and urine (32.6%) [12]. Comparatively, the work of Campos et al. was dominated by strains from these two clinical materials. In addition, a high percentage of strains isolated from BAL (21%) was observed in this study [15]. Similar results were obtained by Li et al., who isolated 16.4% of strains from this material [16]. Our study showed that the highest number of *K. pneumoniae* strains came from urine (46.4%), BAL (18.85%), and recital swabs (14.5%). A study by Aires-de-Sousa et al. isolated a small percentage of strains from blood (8.7%) [12]. An identical result was obtained in our work, wherein 8.7% of isolates also came from this material.

Uncontrolled for many years, the overuse of antibiotics has contributed to one of the greatest threats to public health worldwide. The emergence of multidrug-resistant pathogens, such as *K. pneumoniae*, poses a significant challenge in clinical practice. The reactions of bacteria, allowing them to evade antibiotics, are testament to their excellent adaptation, and are the best evidence of the evolution of these pathogens [17]. Their enormous genetic capabilities result in the emergence of multidrug-resistant and highly drug-resistant strains [18]. Gao et al. observed that all carbapenemase-positive strains of *K. pneumoniae* showed complete resistance to penicillins with inhibitors and cephalosporins [19]. Apondi et al. showed that resistance to cephalosporins was 81.8% [20]. On the other hand, in the work of Durdu et al., it was observed that the percentage of resistance to cefepime and ciprofloxacin in isolates was 91.3% and 72.6%, respectively [21]. On the other hand, in the work of Singh et al., it can be seen that the percentage of resistance to cefotaxime was 70%, to cefepime was 100%, and to ciprofloxacin was 65% [22]. In our study, the percentage of strains resistant to cephalosporins, quinolones, and penicillin with inhibitors was 100%. Apondi et al. found that 21% of strains were resistant to amikacin, and 82.8% to gentamicin [20]. Other results were obtained by Durdu et al., who determined that 55.1% and 55.3% of strains were resistant to these antibiotics, respectively [21]. In our study, 53.6% and 82.6% of isolates were insensitive to amikacin and gentamicin, respectively, indicating that resistance to this group of drugs among *K. pneumoniae* strains is quite diverse.

Clinicians worldwide are significantly concerned about the growing Gram-negative bacilli of resistance to carbapenems, drugs that until recently were the last bastion in the fight against infections. A study by Zheng et al. showed a high resistance of isolates to carbapenems, at 99% for meropenem and 100% for imipenem [23]. Gao et al. presented similar findings, in which all labeled strains were resistant to carbapenems [19]. The percentages of strains resistant to imipenem and meropenem obtained in our study were 81.2% and 76.8%, respectively.

*K. pneumoniae* is a major producer of carbapenemases, classified into the KPC, MBL (NDM), and OXA-48 families [24]. In a recent report, the World Health Organization (WHO) announced that carbapenemase-producing *K. pneumoniae* strains are already present in all regions of the world, in some with a prevalence of more than 50% [18]. Epidemiological studies indicate that particular carbapenemases predominate in different parts of the globe. NDM is the main enzyme found in India, Pakistan, and Sri Lanka. KPC-type carbapenemases are endemic in the United States, China, Australia, South America, Greece, and Italy. In contrast, OXA-48 enzymes are endemic in Turkey, Malta, the Middle East, and North Africa [25]. A paper by Logan et al. estimated that the most commonly isolated carbapenemases in the US are NPC enzymes, while NDM and OXA-48 occur sporadically [26]. Similar conclusions were made by Han et al., who showed that KPC accounts for 52% of enzymes produced by strains in China. NDM-type carbapenemases account for 11%, and OXA-48 accounts for 37% [27]. Kazi et al. showed that 76.57% of strains produced NDM enzymes in India, and 4.5% produced OXA-48 [28]. The results of a study by Lopes et al. conducted in Portugal were characterized by the presence of KPC-type carbapenemases (91%) and OXA-48 (9%) [29]. A study of the epidemic situation in Poland was carried out by Ojdana et al., who found that the primary type of carbapenemases produced by *K. pneumoniae* in our country is NDM-metallo-β-lactamases (71%), and, to a lesser extent, KPC enzymes (29%) [30]. These conclusions were also confirmed by Albigeret al., who showed a decrease in the number of *K. pneumoniae* producing KPC and an increase in the number of *K. pneumoniae* producing NDM [31]. In our study, the percentage of *K. pneumoniae* strains producing NDM-type carbapenemases was also the highest (81.2%). KPC and OXA-48 enzymes accounted for 7.2% and 11.6%, respectively.

Risk factors for *K. pneumoniae* infection are primarily related to healthcare facilities and associated extended hospitalization, overuse of antibiotics, or use of invasive diagnostic tools such as urinary catheters, vascular catheters, and mechanical ventilation. In hospitals, *K. pneumoniae* is easily transmitted from patients to healthcare workers and vice versa. In addition, the microorganism’s ability to survive on the surfaces of hospital equipment facilitates its spread. Successful transmission of *K. pneumoniae* between wards is the cause of intrahospital outbreaks. This is why close surveillance and isolation of carriers of this pathogen is so essential [32]. An important element in reducing nosocomial infections is a thorough understanding of the pathways of the spread of the microorganism and its reservoirs. The PFGE method is considered the gold standard in epidemiological investigation. Using this method, it was confirmed that *K. pneumoniae* isolated from hospitals in different regions of Romania belonged to a single clone and was responsible for causing the outbreak [33]. Wang et al. found that one clone of *K. pneumoniae* isolated from burn wards led to an outbreak in a hospital in China [34]. Another study conducted in China by Kong et al. found clonal relatedness of *K. pneumoniae* NDM strains isolated from a neonatal ward [35]. Mansour et al. identified colistin-resistant isolates that had identical PFGE profiles. The isolation of *K. pneumoniae* strains showing genetic relatedness from patients in different wards of a hospital in Tunisia strongly suggested clonal transmission of a multidrug-resistant strain [36]. By using methods to determine the genetic relatedness of the strains, Duman et al. showed that a *K. pneumoniae* clone coproducing OXA-48 and NDM enzymes led to an outbreak in the intensive care unit of a hospital in Turkey [37]. Chen et al. used the PFGE method not to confirm, but to rule out the possibility of a hospital outbreak caused by a particular clone. They showed that the *K. pneumoniae* strains they analyzed belonged to three genetic groups with similar homology [38]. Our study confirmed that *K. pneumoniae,* classified into genotypes A, B, and C, came from patients hospitalized in the wards of two hospitals (J1 and J2) in one province. This may indicate the epidemic nature of these strains, mainly since J2 patients were transported to J1 for surgery. Additionally, the results of a similarity analysis conducted at a third facility in another province were extremely interesting, wherein all strains were assigned to a single D genotype (21) that was not present in the previously described hospitals. Thus, it can be concluded that *K. pneumoniae* strains have spread between hospitals within a single province, confirming a local epidemic problem. The results underscore the usefulness of the PFGE method in epidemiological investigation and the determination of epidemic outbreaks.

The retrospective nature of the study and its small sample size are inherent limitations of the study. In addition, these are data from a few centers of a particular city, which limits generalizability to other geographic areas or institutions.

## 4. Materials and Methods

### 4.1. Bacterial Strains

We conducted a retrospective multicenter study on 69 *K. pneumoniae* strains belonging to the collection of the Department of Microbiology, Immunology and Laboratory Medicine of the Pomeranian Medical University, Szczecin (Poland). The strains were isolated from patients between Dec 2019 and Feb 2021 at three hospitals (Independent Clinical Hospital No.1 in Szczecin, Independent Clinical Hospital No. 2, Pomeranian Medical University Szczecin, and Independent Provincial Public Integrated Hospital “Zdunowo” in Szczecin). The clinical materials from which the isolates were derived were blood, urine, bronchoalveolar lavage (BAL), pus, rectal swabs, and wound swabs.

### 4.2. Identification and Determination of Drug Susceptibility

*K. pneumoniae* strains were identified to species using VITEK 2 Compact (bioMérieux, Craponne, France). A disk diffusion method was used to assess the drug susceptibility of the isolates. The diameters of the zones of bacterial inhibition around the discs were accurately measured. The values obtained were assigned to a selected sensitivity category based on the recommendations of the European Committee on Antimicrobial Susceptibility Testing (EUCAST) [39].

### 4.3. Determination of Carbapenemases

The growth of *K. pneumoniae* as green colonies on a chromogenic medium (ChromIDCarba, bioMérieux, France) indicated the strains’ ability to produce carbapenemases. The presence of genes encoding carbapenemases was confirmed using Real-Time PCR XpertCarbaR (Cepheid, Solna, Sweden).

### 4.4. Determination of Phylogenetic Similarity of K. pneumoniae Strains by PFGE Method

According to the manufacturer’s instructions, DNA isolation was performed using the CHEF Bacterial Genomic DNA Plug Kit (Bio-Rad, Hercules, CA, USA). Restriction digestion of isolated DNA was carried out using Tango buffer and *XbaI* enzyme (Thermo Scientific, Waltham, MA, USA). A solution was prepared with 1.2 g of agarose (DNA Gdansk, Poland) dissolved in 100 mL of TBE buffer to prepare the agarose gel. Electrophoresis was conducted in 0.5× concentrated TBE buffer at 14 °C for 24 h. After electrophoretic separation, the gel was stained with 0.5 μg/mL ethidium bromide (Sigma-Aldrich, Darmstadt, Germany) for 30 min. A GelDoc-It2 Imager system (Upland, CA, USA) was used to read the results.

## 5. Conclusions

*K. pneumoniae* strains were the most frequently cultured from urine, indicating the high prevalence of UTIs in hospitalized patients. The strains were highly resistant to β-lactams with β-lactamase inhibitors, cephalosporins, and quinolones. A low percentage of isolates showed sensitivity to aminoglycosides and carbapenems. The most commonly produced carbapenemases include enzymes from the metallo-β-lactamases (NDMs) family. *K. pneumoniae* strains were classified into several different genotypic clusters, but the high similarity of isolates was confirmed in departments of the same hospital and between hospitals of the same province.

## Figures and Tables

**Table 1:** Drug susceptibility of *K. pneumoniae* strains.

Antibiotic	Antibiotic Concentration [µg]	Susceptible *n* (%)	Resistant *n* (%)
Amoxicillin with clavulanic acid	20/10	0 (0)	69 (100)
Piperacillin/tazobactam	100/10	0 (0)	69 (100)
Cefotaxime	30	0 (0)	69 (100)
Cefepime	30	0 (0)	69 (100)
Gentamicin	10	12 (17.4)	57 (82.6)
Amikacin	30	32 (46.4)	37 (53.6)
Ciprofloxacin	5	0 (0)	69 (100)
Imipenem	10	2 (2.9)	67 (97.1)
Meropenem	10	1 (1.5)	68 (98.5)

**Table 2:** Occurrence of genotypes among *K. pneumoniae* isolates in individual hospitals.

Genotype	Number of Strains *n* = 69	Hospital
**A**	35	H1 (25), H2 (10)
**B**	3	H1 (2), H2 (1)
**C**	6	H1 (4), H2 (2)
**D**	21	H3
**E**	4	H1

## Data Availability

Not applicable.
